# Arteriovenous Fistula Embolization in Suspected Parauterine Choriocarcinoma

**DOI:** 10.1155/2016/6353471

**Published:** 2016-06-14

**Authors:** Husain Alturkistani, Mohamed-Karji Almarzooqi, Vincent Oliva, Patrick Gilbert

**Affiliations:** ^1^Division of Interventional Radiology, Department of Radiology, Notre-Dame Hospital, University of Montreal Hospital Centers (CHUM), 1560 Sherbrooke East, Montreal, QC, Canada H2L 4M1; ^2^Radiology Department, King Khalid University Hospital, King Saud University, Riyadh 12372, Saudi Arabia

## Abstract

This is a case of choriocarcinoma that did not regress after chemotherapy treatment. A 30-year-old female patient (gravida 2, para 2), presented to our ER with stroke and persistent mild pelvic pain 2 months after a Caesarean section. Computed tomography (CT) revealed an ischemic left hemicerebellar region and a hypervascular mass in the pelvic region. This mass was not present on routine fetal ultrasound during pregnancy. The lesion was treated by chemotherapy after closure of a foramen ovale and insertion of an inferior vena cava (IVC) filter. After that, 2 courses of EMACO (Etoposide, Methotrexate, Actinomycin D, Cyclophosphamide, and Vincristine) chemotherapy regimen were given. Posttreatment CT showed the hypervascular mass without any changes. Arteriography showed the arteriovenous fistulae that were embolized successfully with plugs, coils, and glue. Embolization was considered due to the risk of acute hemorrhagic life-threatening complications. Eight chemotherapy courses were added after embolization. Treatment by endovascular approach and reduction of the hypervascular mass can be a valuable adjunct to chemotherapy treatment of choriocarcinoma.

## 1. Introduction

There are 6 types of gestational trophoblastic disease: complete and partial hydatidiform mole, invasive mole, choriocarcinoma, placental site trophoblastic tumor, and epithelioid trophoblastic tumor (EST) [1]. The incidence of molar pregnancies is about 1 in 1000 pregnancies in Western populations [2]. They can be detected by ultrasound or elevated human chorionic gonadotropin (*β*-hCG) levels [3]. Choriocarcinoma is a rare type of malignant tumor that affects 1 in 40 000 pregnancies and 1 in 40 hydatidiform moles [2]. Choriocarcinoma can present with arteriovenous fistulae and massive uterine bleeding [4]. Our patient presented with stroke and pelvic pain 2 months after a Caesarean section. An abdominopelvic CT scan showed a hypervascular mass in the left pelvic region that was diagnosed, with relatively elevated levels of human chorionic gonadotropin (*β*-hCG), as choriocarcinoma. In this high risk patient, we treated the hypervascular mass by embolization after failure of chemotherapy to decrease its size.

## 2. Case Report

**Figure 1:**
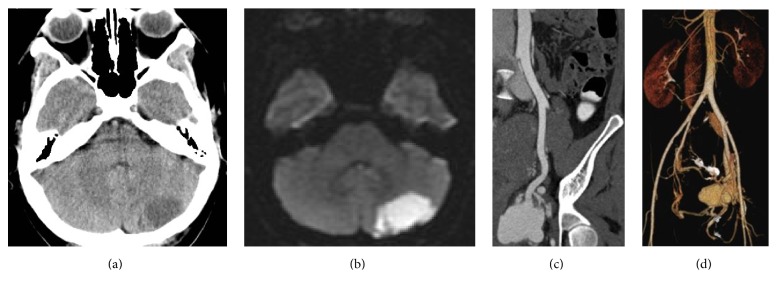
(a) Noninjected head CT with hypodense left hemicerebellar lobe. (b) Brain MRI: diffusion sequence confirming the hyperintense left hemicerebellar ischemic lesion. (c) Coronal oblique view of the pelvic mass in the arterial phase. (d) Volume rendering showing the mass with its arterial supply and venous drainage. Iliac vein thrombosis is not shown in this figure.

A 30-year-old female (gravida 2, para 2) presented with signs of stroke (acute headache, dizziness, loss of coordination, and blurry vision) and a dull aching pelvic pain with heaviness 2 months after a Caesarean section (C/S). Patient thought pelvic pain was normally expected after C/S. On admission, head CT revealed a hypodense area in the left cerebellar hemisphere (Figure 1(a)). Brain MRI done on the same day showed hyperintensity in the diffusion sequence, confirming an ischemic left hemicerebellar lesion (Figure 1(b)). An abdominopelvic CT was performed and showed a hyperdense left hemipelvic parauterine lobular mass (4 × 6 cm in diameter) supplied by the left inferior gluteal artery, left uterine artery, and other small arterial branches. There was also evidence of a left iliac vein thrombus. Patient was transferred to us from a periphery hospital. Due to the recent stroke from a possible embolic event, an echocardiogram was necessary to exclude a patent foramen ovale, which was confirmed and closed on day 5 of patient's hospital stay by the cardiologist with an Amplatz*™* prosthesis (AGA PFO 25 mm prosthesis, St. Jude Medical, USA). As anticoagulation was contraindicated, an IVC filter was placed on day 4 of patient's hospital stay. Blood work revealed no abnormalities with the exception of an elevated human chorionic gonadotropin (*β*-hCG) level, which was at 584 U/L 2 months after Caesarean section. The lesion was diagnosed as choriocarcinoma with FIGO stage IV and high risk score.

Following placement of an IVC filter and treatment of the patent foramen ovale, the patient underwent, starting on day 4 of her hospital stay, 2 cycles of EMACO regimen chemotherapy treatment (Etoposide, Methotrexate, Actinomycin D, Cyclophosphamide, and Vincristine). A decreased *β*-hCG level was observed (382 U/L) without any regression of the vascular mass. The hypervascular lesion revealed many feeding arterial branches and early draining veins returning to the left common iliac vein and then to the inferior vena cava (IVC). Based on this type of enhancement and the characteristics of the lesion, it was classified as an arteriovenous fistula (type II arteriovenous malformation). As it was a high risk lesion with atypical presentation of choriocarcinoma, embolization was considered 5 weeks after patient's admission, as an adjunct to chemotherapy.

**Figure 2:**
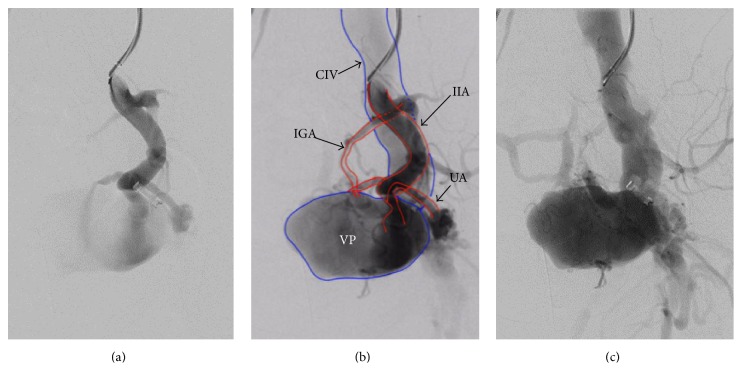
Digital subtraction angiography (DSA) of the pelvic mass. (a) Catheter in the left internal iliac artery. (b) Illustration of the arteriovenous fistula: “red: arterial feeding branches; blue: venous pouch and left common iliac vein; IIA: internal iliac artery; IGA: inferior gluteal artery; UA: uterine artery; CIV: common iliac vein; VP: venous pouch.” (c) Late arterial phase with rapid opacification of the left common iliac vein.

**Figure 3:**
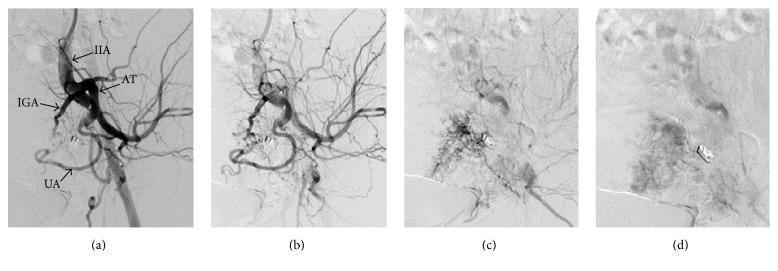
DSA after embolization. (a) Arterial phase: “IIA: internal iliac artery; IGA: inferior gluteal artery; UA: uterine artery; AT: anterior trunk of internal iliac artery.” (b) Delayed arterial phase without venous opacification. (c) Early venous phase with minimal venous pouch opacification. (d) Late venous phase with no direct opacification of the venous pouch from the fistula.

Access was gained by a right common femoral artery puncture and a 6 Fr introducer sheath was inserted. A 4 Fr Cobra catheter was used to catheterize the left internal iliac artery and the ensuing angiography revealed multiple feeding arterial branches (principally, the inferior gluteal artery and the uterine artery) in a venous draining pouch via direct fistulae (Figure 2). The 6 Fr sheath was exchanged for a 45 cm long 7 Fr sheath (Destination*™*, Terumo, Japan) that was positioned in the left internal iliac artery. The feeding branches were catheterized with a 4 Fr Cobra catheter and 0.035 Terumo guidewire. The three main feeding branches came from the inferior gluteal artery and were occluded at its venous connection with 6 and 8 mm diameter 4th-generation Amplatzer*™* plugs (St. Jude Medical, USA). Another branch came from the left uterine artery and was occluded with an 8 mm diameter 2nd-generation Amplatzer plug (St. Jude Medical, USA). Two small branches from the posterior trunk of the left internal iliac artery with a direct flow into the venous pouch were catheterized with a microcatheter (Marathon*™*, Covidien, USA) on microguidewire (X-Pedion*™*, Covidien, USA) and embolized with a detachable 3 mm Interlock*™* coil (IDC*™*, Boston Scientific, USA). Other smaller branches were embolized with glue (TissueSeal, USA). A postembolization angiogram showed satisfactory results with only one microbranch perfusing the pouch. However, no early draining veins were identified (Figure 3). *β*-hCG levels had dropped to 103 U/L 2 days after embolization and 12 U/L 2 weeks after embolization and had completely returned to normal (<0.6 U/L) 5 months after embolization (normal *β*-hCG < 5 U/L).

**Figure 4:**
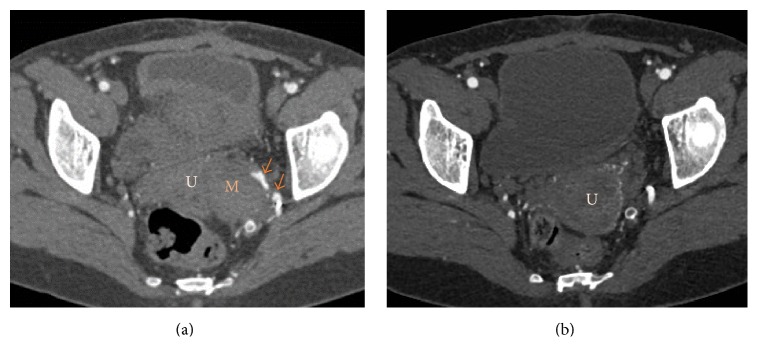
Follow-up pelvic CT scan. (a) CT scan 3 weeks after embolization: presence of some microfistulae (red arrows) and complete obscuration of the venous pouch without regression of mass size. (b) CT scan 1 year after embolization shows complete regression of the mass without any suspicious residual tissue (U = uterus, M = mass).

A follow-up pelvic CT scan 3 weeks after procedure showed venous thrombosis of the aneurysmal pouch and one persistent microfistula. However, there was no evidence of early venous drainage. Tumor size was not decreased. Clinically, the patient was improving. Pelvic CT and MRI performed after one year showed complete thrombosis without any suspicious residual tissue (Figure 4).

## 3. Discussion

Choriocarcinomas can occur subsequently with normal or ectopic pregnancies. These tumors are highly vascular lesions [5]. They originate from the placenta in women due to retained trophoblastic tissue in the uterus after placental expulsion and disseminate through vascular routes. These tumors can therefore be seen in various locations, such as the uterine cervix, Fallopian tubes, ovaries, vagina, vulva, and sometimes extragenital organs, such as the liver, lungs, and brain [6]. Depending on the time of appearance, they can be classified as early or late. A lesion found after menopause is considered late type due to trophoblastic cells that remain dormant for a long time and become active afterwards [5]. Pelvic arteriovenous fistulae are rare complications of gestational trophoblastic disease [7].

In this case, our patient presented with early-type choriocarcinoma that developed soon after her Caesarean section. The stroke alerted us to the risk of further embolic events. For this reason, the foramen ovale was treated by the cardiologist and an IVC filter was inserted. Biopsy should be avoided in suspicious cases due to the risk of bleeding. After these precautions, the patient received a full dose of chemotherapy, as this is the treatment of choice [8].

Finding persistent arteriovenous fistulae after successful chemotherapy of malignant trophoblastic disease (choriocarcinomas) is not uncommon [9]. In this case, treatment was successful in reducing *β*-hCG levels, but tumor size was not affected. Embolization was considered due to the risk of acute hemorrhagic life-threatening complications. Low *β*-hCG titer in this case could be interpreted by atypical presentation of choriocarcinoma with mainly AVF component predominating the clinical picture. In the meanwhile, we could not diagnose it as phantom choriocarcinoma as *β*-hCG level was not that low [10].

Arteriovenous fistula embolization can be done using different materials, such as plugs, coils, glue, alcohol, and onyx [11]. In this case, the mass was embolized using plugs, coils, and glue. This mass corresponds to an arteriovenous malformation (AVM) with many feeding arterial branches and a single venous pouch, type II AVM [11]. The large feeding branches were closed with plugs, while the small feeding branches were closed with coils and glue. Glue was only used in small branches with decreased flow to maintain control of embolization. The goal of embolization was to reduce vascular supply and achieve thrombosis of the venous pouch.

In conclusion, when treatment of choriocarcinoma with chemotherapy does not reduce the size of the hypervascular mass, we recommend the possibility of treating the mass by endovascular methods. Embolization should be considered in cases of choriocarcinoma associated with arteriovenous fistulae with a risk of acute hemorrhagic life-threatening complications.
